# Case report: Hysteroscopy combined with a vaginal mold for severe recurrent vaginal adhesion and stenosis with pyocolpos after pelvic fracture in a 13-year-old female

**DOI:** 10.3389/fped.2022.966724

**Published:** 2022-10-21

**Authors:** Jiao Wang, Fei Zheng, Dandan Wang, Qing Yang

**Affiliations:** Department of Obstetrics and Gynecology, Shengjing Hospital of China Medical University, Shenyang, China

**Keywords:** Vaginal adhesion, obstructive symptoms, pediatric pelvic fracture, hysteroscopy, vaginal dilation

## Abstract

Vaginal adhesions and stenosis are infrequent long-term sequelae following pediatric pelvic fractures. Patients may not present with signs and symptoms before menarche, leading to delayed diagnosis and treatment. We report an adolescent girl who presented with a post-abdominal trauma pelvic fracture and urethrovaginal fistula and subsequent severe vaginal adhesion, which resulted in infection and obstructive symptoms after menarche. Hysteroscopy-guided vaginal adhesion release using an epidural catheter and ultrasonography was performed, followed by vaginal dilation, to resolve obstructive symptoms. For girls with pelvic fractures, education on possible long-term sequelae is required, as is regular follow-up. Timely diagnosis and treatment are important, and hysteroscopic release of vaginal adhesion and postoperative regular vaginal dilation may be an effective treatment.

## Introduction

Pediatric pelvic fractures are often minimally or non-displaced; however, significant co-existent intraabdominal injuries can occur, most commonly in the liver, spleen, and genitourinary system (1). Urethral injury following a pelvic fracture is common and easily recognized; however, long-term sequelae from vaginal injuries after pelvic fracture, such as vaginal adhesions and stenosis, in children are infrequent and may not be recognized and treated in a timely manner. Here, we present the diagnosis and treatment of recurrent vaginal adhesions and stenosis in an adolescent girl who had sustained a pelvic fracture 3.5 years prior to presentation at our hospital outpatient department in 2019. This patient underwent vaginal adhesion release under hysteroscopy using an epidural catheter and ultrasonography, followed by regular vaginal dilation to successfully treat her obstructive symptoms.

## Case report

On May 10, 2019, a 13-year-old girl presented to our hospital with a 1-year history of dysmenorrhea. On November 15, 2015, she sustained a traumatic abdominal injury due to the collapse of a wall that resulted in a pelvic fracture and small intestine rupture. She subsequently underwent emergency partial resection and anastomosis of the small intestine at a local hospital. Intraoperatively, a gynecologist observed normal uterus and bilateral adnexa, and no rupture or bleeding. A small volume of pink blood was detected in the vagina, but no active bleeding was observed. The pink blood persisted for 8 days, after which intermittent abnormal urine leakage was identified at the vaginal orifice. Due to ineffective conservative treatment, transabdominal urethrovaginal fistula repair and cystostomy were performed at our hospital on July 6, 2016. Preoperative urinary tract ultrasonography showed hydrocolpos (size, 3.1 cm × 3.0 cm × 1.9 cm). Intraoperatively, a urethrovaginal fistula (diameter, approximately 5 mm) was discovered behind the normal internal urethral orifice. A no. 8 silicone catheter was placed into the vagina to confirm the absence of vaginal damage. The intraoperative and postoperative periods were uneventful.

**Figure 1:**
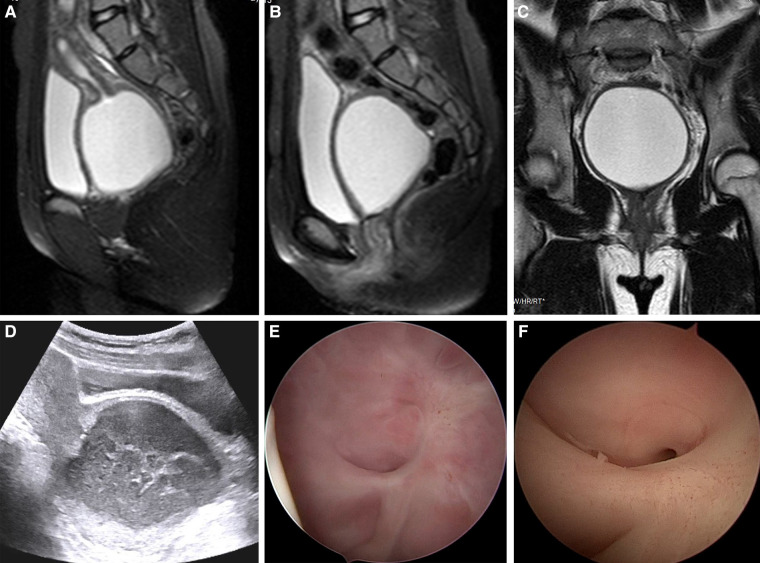
Imaging findings and outpatient hysteroscopic images of the patient. (**A–C**) Pelvic MRI showed a large amount of effusion in the middle and upper segments of the vagina and that the lower segment of the vagina was adhered to the bladder neck. (**D**) Pelvic ultrasound showed hydrocolpos (range, approximately 7.5 cm × 7.2 cm × 6.5 cm) with a flocculent echo. (**E,F**) Outpatient hysteroscopy revealed a small hole at the top of the vagina, which was blocked by a transverse fold, and the cervix was not observable.

In May 2018, she experienced menarche. Five months later, she visited a local hospital complaining of dysmenorrhea, and vaginal adhesion and hematocolpos were identified. She underwent vaginal adhesion release at the same local hospital; however, her dysmenorrhea symptoms did not improve postoperatively and gradually worsened. On May 10, 2019, she presented to our hospital outpatient department and underwent pelvic magnetic resonance imaging (MRI) that revealed a large effusion in the middle and upper segments of the vagina and the lower segment of the vagina as being adhered to the bladder neck (Figures 1A–C). One week later, retrograde urography revealed no urethral or bladder abnormalities. On June 4, 2019, outpatient hysteroscopy showed normal vulva development. The hysteroscope was inserted into the vagina approximately 4 cm from the edge of the hymen and revealed a blind end at the top of the vagina; the cervix was not observable. Therefore, vaginal atresia was diagnosed. On July 8, 2019, she was admitted to our department because of menstrual pain and fever. Her body temperature was 39.8 °C on admission. Blood laboratory test findings were as follows: leukocyte count, 13.6 × 10^9^/L; neutrophil percentage, 85.2%; and C-reactive protein level, 4.01 mg/L. Empirical antibiotic treatment was administered, and a blood bacterial culture tested positive for *Bacteroides fragilis*. Transabdominal pelvic ultrasonography revealed normal uterus and bilateral adnexa, and hydrocolpos (range, approximately 7.5 cm × 7.2 cm × 6.5 cm) with a flocculent echo (Figure 1D). A small amount of vaginal menstrual blood discharge indicated an incorrect diagnosis of vaginal atresia, and an outpatient hysteroscopy was repeated on July 9, 2019. A small hole, which was obstructed by a transverse fold and not easily visible (Figures 1E,F), was identified. Given the patient's medical history, we diagnosed vaginal adhesion resulting in menstrual blood retention and infection. The patient's guardian requested surgical treatment. As she was pubertal and only 13 years old, her reproductive organs were immature; therefore, we recommended surgery to release the adhesion and relieve the obstruction. The patient's guardian consented to our treatment plan. Sexually active patients or those with mature organs may require alternative treatment options for vaginal stenosis.

**Figure 2:**
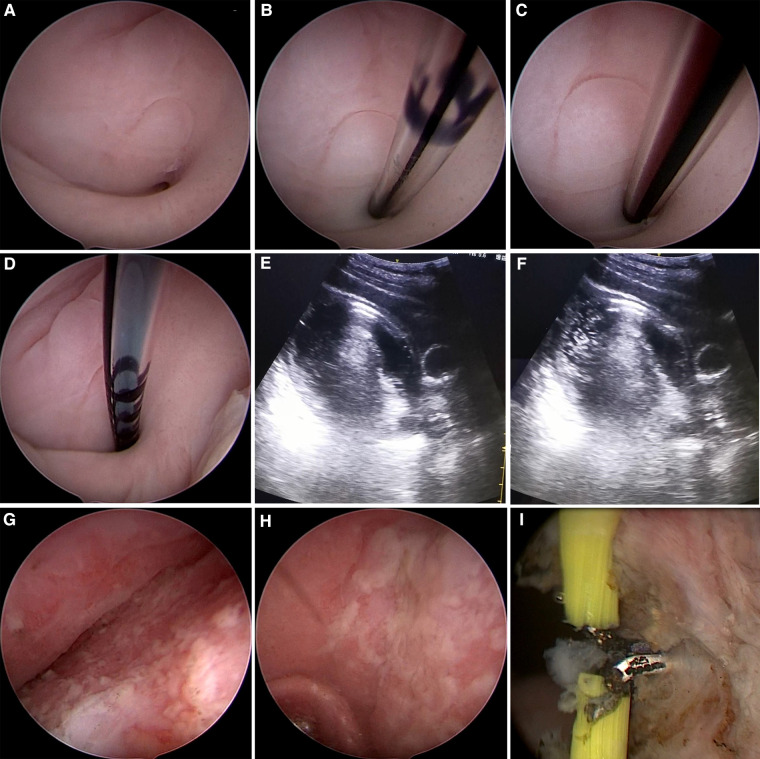
Hysteroscopic and ultrasonic images of the patient during operation. (**A**) A small hole with a diameter of approximately 1.5 mm was seen at approximately 6 o’clock of the top of the vagina. (**B**) An epidural catheter was inserted through the hole. (**C**) Dark red fluid was observed on suction. (**D**) An injection of 20 ml methylene blue was administered through the epidural catheter. (**E,F**) A fluid flow shadow in the vaginal mass was observed after injection of methylene blue under ultrasonography. (**G,H**) A large amount of sloughy pus was observed attached to the vaginal wall and cervix. (**I**) A needle electrode was used to release the adhesion to the vaginal wall longitudinally.

On July 11, 2019, the patient underwent hysteroscopic exploration under general anesthesia. Under ultrasonic monitoring, an effusion of approximately 8 cm × 7 cm was observed in the middle and upper segments of the vagina. A 5-mm Bettocchi hysteroscope (Storz, Germany) was placed and gradually inserted into the vagina for approximately 4 cm to reach a nearly blind end. After careful exploration, a small hole with a diameter of approximately 1.5 mm was seen at approximately the 6 o'clock position (Figure 2A), and the hysteroscope could not be advanced further. An epidural catheter was inserted through the hole using the 5 Fr operative channel of the hysteroscope (Figure 2B), and dark red fluid was observed on suction (Figure 2C). Methylene blue (20 ml) was then administered through the epidural catheter (Figure 2D), and a fluid flow shadow in the vaginal mass was observed under ultrasonography (Figures 2E,F). A Hegar cervical dilator was used to gradually dilate the hole to no. 7. During dilation, a large quantity of malodourous, dark red, purulent fluid flowed out from inside the hole. After sufficient flushing, the hysteroscope was re-inserted, and on entering the upper segment of the vagina, a large amount of sloughy pus was observed attached to the vaginal wall and cervix (Figures 2G,H). After flushing out the pus and further dilating the hole, a 10-mm resectoscope with an electrical needle was used to longitudinally release the vaginal wall adhesion with 80 W power (Figure 2I). A no. 2 absorbable suture was used to stem the bleeding and smooth the vaginal mucosa over the rough surgical wound to reduce re-adhesion. The vagina was plugged with petrolatum gauze, and the surgery was completed. Postoperatively, antibiotic therapy was continued to treat the infection, and her body temperature normalized on day 3. The vagina was disinfected and the petrolatum gauze plug was changed daily. Until postoperative day 5, a mold was used to expand the vagina. The patient was instructed to insert the mold to manually dilate the vagina each day and to place the small-sized mold into the vagina throughout the day and at night for the first month. A medium-sized mold was then used only at night for the following 2 months. The patient recovered well and was discharged on postoperative day 6.

**Figure 3:**
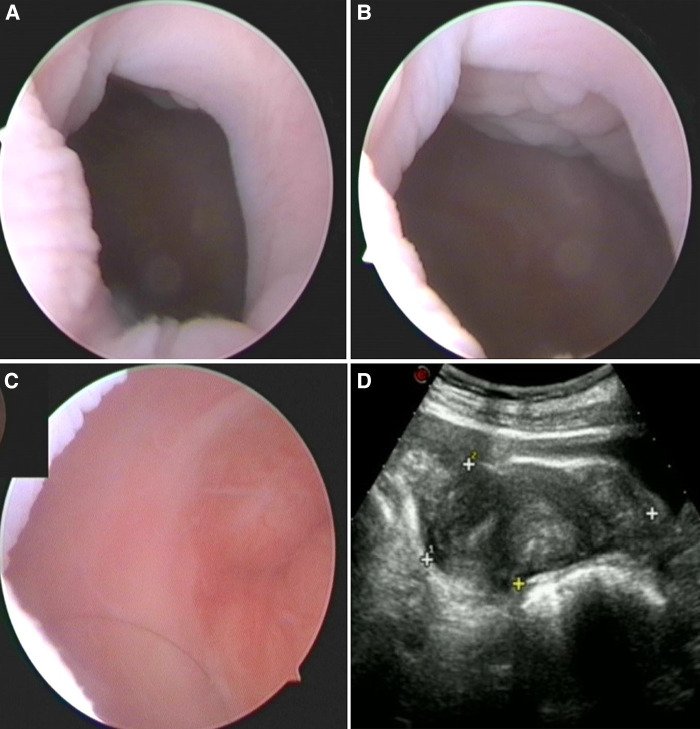
Outpatient hysteroscopic and ultrasonic images 3 months after operation. (**A,B**) Hysteroscopy showed that the surgical wound healed well. (**C**) The cervix could be observed. (**D**) A pelvic ultrasound showed no abnormalities.

Three months postoperatively, hysteroscopy showed a well-healed surgical wound (Figures 3A,B) and identifiable cervix (Figure 3C). Pelvic ultrasonography showed no abnormalities (Figure 3D). At 12 months follow-up, our patient had recovered well; therefore, the use of the mold was stopped. To date, she has no dysmenorrhea symptoms.

## Discussion

Vaginal stenosis or gynatresia is often congenital, occurring as part of the Mayer-Rokitansky-Kuster-Hauser syndrome (2). Acquired vaginal adhesion and stenosis are not commonly observed clinically, especially in children. Acquired vaginal adhesion, stenosis, and atresia may be due to the following: insertion of caustic vaginal pessaries or herbs (3), injury during the birthing process (4), chemical burns to the vagina (2), chemical vaginitis (5), long-term vaginal foreign body (6), vaginal or transvaginal surgery (7, 8), radiotherapy (9), pelvic fracture (1, 10), and chronic graft-vs.-host disease (11). Vaginal foreign bodies and pelvic fractures are the main causes of acquired vaginal adhesions and stenosis in children and lead to sequelae after vaginal injury.

The long-term sequelae of vaginal injury may not be recognized in a timely manner as a considerable time interval may occur between an initial injury and the onset of symptoms. Obstructive uterovaginal anomalies may present after puberty with amenorrhea, dysmenorrhea, pelvic pain, recurrent vaginal discharge, and infertility (12). Vaginal obstruction and menstrual blood accumulation, caused by vaginal adhesion and severe vaginal stenosis, may result in hematometra or hematocolpos with concurrent infection. Differentiating between vaginal adhesion and congenital genital malformations, such as imperforate hymen, transverse vaginal septum, and oblique vaginal septum, is necessary in children.

Patient evaluations for a suspected obstructive reproductive anomaly should include a detailed medical history and physical and imaging examinations (12). Our patient had sustained a pelvic fracture and small bowel rupture due to abdominal trauma and a small volume of vaginal blood was observed intraoperatively; however, a urethrovaginal fistula was not diagnosed. Postoperatively, intermittent vaginal leakage was observed and urethrovaginal fistula repair surgery was then performed after >7 months of ineffective conservative treatment. Preoperative urinary tract ultrasonography revealed hydrocolpos, indicating vaginal adhesion. Despite using a silicone catheter during the surgical repair to avoid vaginal injury, the vagina was not carefully examined. A diagnosis of vaginal adhesion was not confirmed until the patient experienced periodic pelvic pain after menarche. A delayed diagnosis is mainly associated with an asymptomatic period before the accumulation of menstrual blood. Imaging examinations such as ultrasonography and MRI have limitations in distinguishing among vaginal adhesion, atresia, and septum, especially when hematocolpos is absent. Our patient was 13 years old; therefore, we chose to perform hysteroscopy without a speculum to evaluate the degree and location of vaginal adhesion and stenosis. However, the small obscure hole was not identified during her first outpatient hysteroscopy until the second hysteroscopy after admission.

Adolescents may not be sufficiently mature or equipped to adhere to a postoperative dilation schedule; therefore, obstetrician–gynecologists and other gynecologic care providers should consider menstrual suppression and delay of surgical intervention, until a patient is able to perform postoperative vaginal dilation (12). Our patient and her guardian were educated on the possible methods of treatment and their prognosis. As our patient had previously undergone a urethrovaginal fistula repair, vaginal surgery may have increased the risk of fistula recurrence and damage to her surrounding organs; however, her obstructive symptoms due to vaginal adhesion and stenosis resulted in serious physiological and psychological impacts. In addition to her intolerable pelvic pain during menstruation, the infection and pyocolpos were malodourous, and she was unable to lead a normal school life. Our patient and her guardian expressed a strong desire for surgery to resolve her symptoms. We considered it insufficient to inhibit her menstrual cycle, and treatment using vaginal dilators alone was also inappropriate because the hole was extremely small and the dilator was unable to pass through. For our patient, the use of surgical methods to release adhesion and relieve her obstructive symptoms was essential.

Because of the high risks of injury to the bladder, urethra, and rectum when performing this surgery, surgeons should proceed with caution. To confirm that the small hole was connected to the middle and upper segments of the vagina, we intraoperatively inserted an epidural catheter and aspirated the hydrocolpos using a syringe. Simultaneously, we injected methylene blue from the epidural catheter and observed the change of fluid flow shadow in the vaginal mass under ultrasound monitoring. Then we used a cervical dilator to gradually expand the hole, drain the liquid, and flush the pus adequately until the hysteroscope could be inserted. Intrauterine adhesions are usually treated using cold instruments to reduce relapse (13). In contrast, vaginal septum resection is performed using electrical instruments during resectoscopic surgery (14). However, as we did not have hysteroscopic cold scissors, we used a needle electrode to sever the adhesions rather than a common cold knife as resectoscopy provided the operator with a clearer surgical field and the possibility to perform more precise operations and also improved hemostatic effect.

Postoperative vaginal dilation is critical to the success of vaginal reconstructive surgery. In the postoperative period, vaginal molds and dilators help decrease scarring and stenosis at the surgical site and improve the vaginal caliber. Postoperatively, petrolatum gauze was used in the short term for vaginal tamponade to prevent adhesion recurrence. When the surgical wound had mostly healed, we instructed the patient to use the vaginal mold to prevent adhesion recurrence, and the mold diameter was gradually increased. Appropriately guided regular and intensive postoperative dilator therapy may decrease the probability of re-adhesion. At the 3-, 6-, and 12-month follow-ups, the patient's dysmenorrhea symptoms had completely resolved, and no abnormality was detected on pelvic ultrasonography; therefore, the mold was no longer required. While her vagina remained slightly narrower than normal, menstrual blood drainage and obstruction relief were achieved. Follow-up visits continue to be scheduled and, should she experience any difficulties with sexual intercourse in adulthood, vaginal dilation or surgery may then be reconsidered.

We reviewed relevant published literature and identified two case reports of vaginal stenosis that developed following a pediatric pelvic fracture. One report involved a 26-year-old female who had sustained a pelvic fracture in a motorcycle accident at the age of 10 years, and who presented with vaginal stenosis precluding sexual intercourse. This patient menstruated through a fistula in the distal vaginal vault. Radiographic and dye studies showed almost complete vaginal obstruction with heterotopic ossification (1). The other reported case involved a 14-year-old girl with primary vaginal calculi, secondary to a urethrovaginal fistula and vaginal stenosis resulting from a pelvic fracture 11 years previously. Initial surgical urethral realignment and anterior colporrhaphy were unsuccessful, and the patient experienced continuous urinary incontinence over an 11-year period. Finally, surgeries were performed to remove the calculi, repair the urethrovaginal fistula, and treat the vaginal stenosis (10). Including our case, all three cases involved a history of pediatric pelvic fracture. Moreover, the diagnosis and treatment of vaginal adhesions and stenosis in all three cases occurred several years after having sustained the pelvic fractures.

There were some shortcomings concerning the diagnosis and treatment of our patient. First, her initial outpatient hysteroscopy examination at our hospital did not reveal the small, obscured hole in the vagina, which led to the second hysteroscopy. Second, the patient was febrile prior to admission. Although antibiotics had been prescribed, her preoperative temperature did not normalize, and there was a risk of infection diffusion intraoperatively. However, had the vaginal obstruction remained untreated, the infection may have been challenging to eradicate.

## Conclusions

For girls with pediatric pelvic fractures, long-term sequelae may involve vaginal adhesion and stenosis, which may then result in obstructive manifestations after menarche, such as dysmenorrhea, periodic pelvic pain, and infection. These vaginal injury sequelae may not be recognized and treated in time because of a time lapse between an initial injury and the onset of symptoms. Doctors need to educate patients and their guardians concerning the possibility of long-term sequelae and regularly follow-up these patients. Once vaginal adhesion and stenosis have been identified, timely treatment is needed to avoid serious physical and psychological complications. Treatment includes surgical and non-surgical methods, and a tailored treatment plan needs to be formulated for each patient. For patients who select a surgical approach, hysteroscopic vaginal adhesion release under ultrasound monitoring combined with postoperative dilation therapy may provide an effective treatment outcome.

## Data Availability

The original contributions presented in the study are included in the article/Supplementary Material, further inquiries can be directed to the corresponding author/s.
